# Utility of Augmented Reality Glasses With Waveguide Optics and Facial Recognition Technology for Patient Verification in a Simulated Outpatient Setting: Physician Questionnaire Survey Study

**DOI:** 10.2196/95664

**Published:** 2026-10-02

**Authors:** Ryo Andy Ogasawara, Soichiro Yoshida, Kentaro Okamoto, Eiki Nagaoka, Hirotaka Sagawa, Kazutaka Sumita, Hajime Tanaka, Satoshi Shiraga, Tomohiro Amemiya, Yasuhisa Fujii

**Affiliations:** 1 Department of Urology, Institute of Science Tokyo, 1-5-45 Yushima, Bunkyo-ku, Tokyo, 113-8519, Japan, 81 3-5803-5295, 81 3-5803-5295; 2 Department of Pediatric Surgery, Institute of Science Tokyo, Bunkyo-ku, Tokyo, Japan; 3 Department of Cardiovascular Surgery, Institute of Science Tokyo, Bunkyo-ku, Tokyo, Japan; 4 Department of Endovascular Surgery, Institute of Science Tokyo, Bunkyo-ku, Tokyo, Japan; 5 Cellid, Inc, Minato-ku, Tokyo, Japan; 6 Department of Electrical and Electronic Engineering, Institute of Science Tokyo, Meguro-ku, Tokyo, Japan

**Keywords:** augmented reality, automated facial recognition, smart glasses, data display, patient care

## Abstract

**Background:**

Augmented reality (AR) glasses with waveguide optics and facial recognition may enhance patient identification and clinical workflows. However, their usability among physicians has not yet been evaluated.

**Objective:**

The objective of this study was to assess the usability and acceptability of prototype AR glasses with facial recognition in a simulated outpatient setting.

**Methods:**

In May 2025, 14 urologists from a single institution tested prototype AR glasses developed by Cellid Inc. The device incorporates waveguide-based optical displays, a miniature camera, and deep learning–based facial recognition software. Physicians identified preregistered mock patients, with demographic and clinical data displayed on the right lens. After the trial, participants completed an anonymous questionnaire on usability, comfort, visibility, and self-reported symptoms using 3- or 5-point Likert scales.

**Results:**

Eleven participants (79%; 95% CI 52%‐92%) found the device helpful in performing clinical procedures, and 12 (86%; 95% CI 60%‐96%) believed it improved patient safety. Most participants adapted quickly to wearing the device, and 79% (95% CI 52%‐92%) expressed a willingness to use it in future practice. Four of 13 respondents (31%; 95% CI 13%‐58%) selected “somewhat tired” for eye fatigue, and only 1 participant experienced minor and transient physical symptoms including headache and nausea. Three of 13 respondents (23%; 95% CI 8%‐50%) rated the glasses as slightly heavy, and among the 5 participants who wore prescription glasses, 4 (80%; 95% CI 38%‐96%) reported difficulty wearing the prototype over their glasses. Surrounding-environment visibility and perceived facial recognition responsiveness were generally favorable; however, 8 of 13 respondents (62%; 95% CI 36%‐82%) rated the displayed patient information as slightly difficult to see.

**Conclusions:**

In this pilot study, AR glasses with facial recognition were well accepted by physicians. This technology has demonstrated perceived clinical utility and has the potential to assist medical professionals, though further refinement and larger clinical evaluations are needed.

## Introduction

Augmented reality (AR) is a technology that overlays digital content onto the real world in real time, thereby enhancing human perception of reality. AR technologies are advancing rapidly in many fields, and the use of AR in clinical settings is highly anticipated. Previous studies have proposed various clinical applications of AR and virtual reality, such as surgical navigation and educational uses for medical students and residents [1-5]. The use of wearable devices in clinical settings requires hardware development and application implementation specifically designed for clinical use, which differs from consumer applications.

Patient misidentification is a critical issue in health care settings. Since patient identification errors can lead to serious medical malpractice, medical staff have to exercise the utmost caution when identifying patients. The accuracy of facial recognition technology is rapidly improving, and its application to patient identification shows great promise. By incorporating the latest AR and facial recognition technologies, health care workers will be able to identify patients more easily and accurately than is currently possible.

A previous study reported favorable overall usability of AR glasses equipped with facial recognition functionality among nurses working in an ophthalmic ward [6]. However, no previous studies have investigated the usability of AR glasses equipped with facial recognition functionality among doctors.

The purpose of this study was to evaluate the clinical usability of AR glasses that are equipped with facial recognition technology developed by Cellid Inc and that use a waveguide-based optical system. This evaluation was conducted through a questionnaire survey targeting physicians in a simulated outpatient scenario. The study was not designed to estimate face-matching accuracy or to determine effects on misidentification, patient safety, or clinical workflow.

## Methods

### Study Design

This study was conducted in May 2025 to evaluate the usability of AR glasses equipped with facial recognition technology in a simulated clinical setting. Urological physicians were invited to wear the AR device and experience its patient identification function using mock patients preregistered in the system. The pilot study used a convenience sample of urologists because urology outpatient practice was the initial formative use case at the investigators’ institution. Participants were tasked with identifying preregistered mock patients using the facial recognition capabilities of the AR device.

### Recruitment and Participants

A total of 14 physicians, including 9 urology specialists and 5 residents, were recruited from the Department of Urology at the Institute of Science Tokyo. All participants used the AR glasses under standardized conditions using mock patient data.

### Device and System Description

The AR glasses used in this study were a see-through type with waveguide optics and weighed 58 g. The device included a miniature camera (Figure 1) mounted on the bridge and required a wired connection to a smartphone (ROG Phone 7, ASUS), weighing 239 g, via a 20 g cable, resulting in a total carry weight of 317 g. Since the prototype AR glasses require external power and do not support wireless data transmission, they must be physically tethered to a smartphone running facial recognition software.

**Figure 1.**
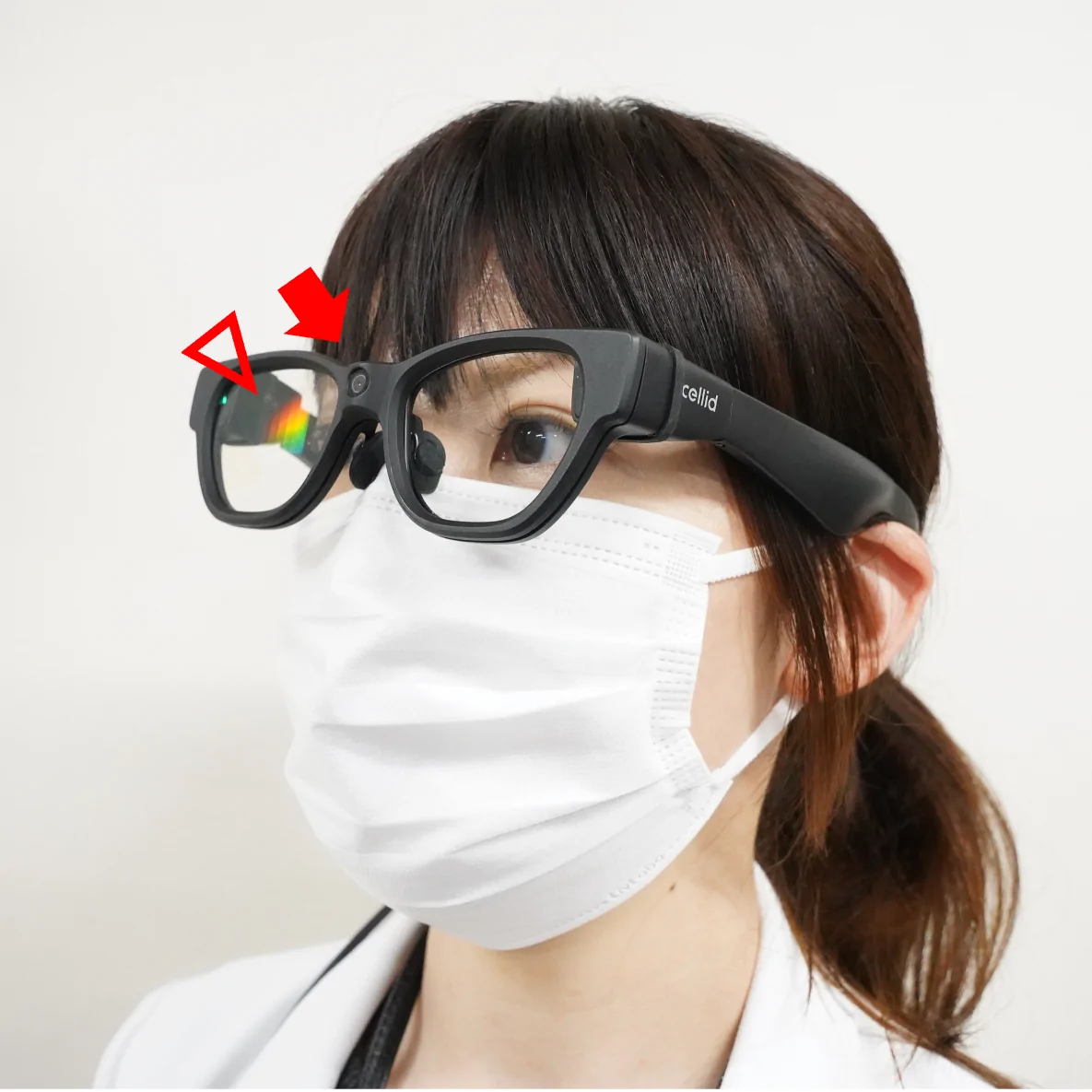
Photograph of a doctor wearing augmented reality glasses. A diffraction grating is installed on the right lens (framed red arrowhead), and a built-in miniature camera is located on the bridge (red arrow).

The facial recognition system was developed by Cellid Inc and used Amazon Rekognition (Amazon Web Services) for deep learning–based image recognition. Upon recognition of a registered face, patient information—including name, age, diagnosis, and allergies—was displayed in real time on the right lens via the waveguide optical system (Figure 2). This design enabled simultaneous visualization of the real-world environment and patient data. During the evaluated task, the display operated as an automatic, 1-way information presentation; direct manipulation of holographic content was not evaluated. Since the pilot study was designed as a user-experience demonstration rather than a technical validation study, recognition attempts, false matches, false rejections, repeated attempts, and software or network failures were not prospectively logged. Consequently, sensitivity, specificity, error rates, recognition success rates, and processing latency cannot be calculated from this study.

**Figure 2.**
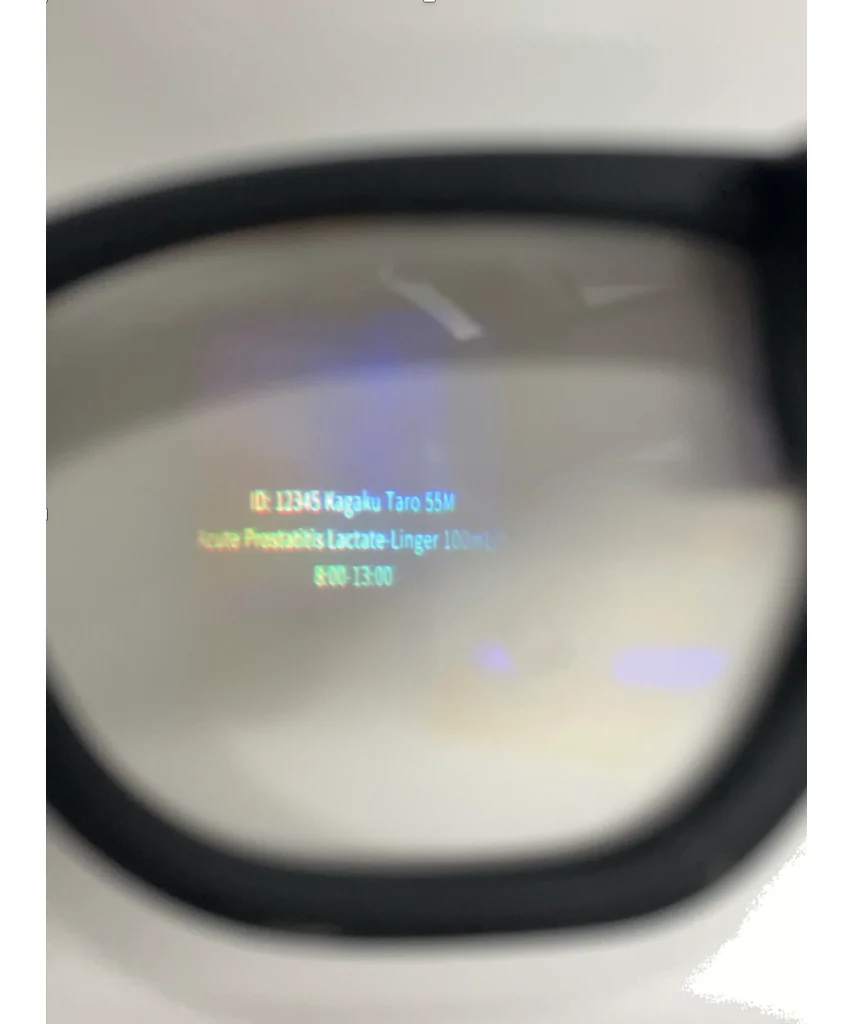
Patient information displayed on the augmented reality glasses lens.

### Data Collection

After using the AR glasses, participants completed an anonymous questionnaire evaluating the glasses’ usability and potential adverse effects. The investigator-developed questionnaire consisted of 15 items and an optional free-text field. The questionnaire was designed to assess the integrated prototype experience rather than facial recognition accuracy alone. Six domains were evaluated: perceived clinical utility (items 1‐2), adaptation and intended adoption (items 3‐4 and 10), wearing-related symptoms (items 5‐9), weight and fit (item 11 and free-text comments), visual usability and task interference (items 12‐14), and perceived facial recognition responsiveness (item 15). Items 1‐11 and 15 used 5-category response scales, while items 12‐14 used 3-category scales. A nonresponse was treated as missing data rather than a scale category. The questionnaire was not psychometrically validated, and no composite usability score was calculated. The complete questionnaire is provided in Multimedia Appendix 1.

### Statistical Analysis

Questionnaire data were analyzed using descriptive statistics. Categorical variables, including responses on usability, comfort, visibility, and adverse effects, are presented as frequencies and percentages. Ordinal variables collected via 3- or 5-point Likert scales are treated as categorical and summarized using counts and percentages for each response category. Continuous variables, such as participants’ years of clinical experience, are reported as median and range. For all reported categorical outcomes, post hoc Wilson score 95% CIs were calculated to show sampling uncertainty. No hypothesis testing, subgroup comparisons, or adjustment for multiplicity was performed because of the small exploratory sample.

### Ethical Considerations

This study was approved by the institutional review board of the Institute of Science Tokyo (approval number: I2024-070). All participants provided written informed consent prior to participation. This study used mock patient data to avoid the risks associated with handling real patient information, thereby minimizing ethical concerns related to privacy and data protection.

## Results

### Principal Findings

Fourteen urology specialists or residents with a median of 13 years of experience as physicians (range 3‐24 years; IQR 3.75-17.75 years) were enrolled in this study. Thirteen (93%) participants were male and 1 (7%) was female. Five (36%) participants wore prescription glasses. All participants used the facial recognition function and completed the anonymous questionnaire. The results of the questionnaire are shown in Table 1.

**Table 1.** Results of questions.

Domain	Questions	Responses (N=14), n
Perceived clinical utility	Did the use of the AR^a^ glasses assist you in performing medical procedures?	Not at all, 0 Slightly unhelpful, 0 Neutral, 3 Slightly helpful, 5 Helpful, 6
Perceived clinical utility	Did the use of the AR glasses help improve safety in clinical practice?	Not at all, 0 Slightly unhelpful, 0 Neutral, 2 Slightly helpful, 3 Helpful, 9
Adaptation and adoption	Did it take time for you to get used to wearing the AR glasses?	Not at all, 6 Minimal, 2 Neutral, 1 Somewhat, 4 Very much, 1
Adaptation and adoption	Do you wish to use AR glasses in future medical procedures?	Not at all, 0 Unlikely, 0 Neutral, 3 Somewhat, 4 Strongly, 7
Self-reported symptoms	To what extent did you experience eye fatigue from using the AR glasses?	Not at all, 5 Minimal, 2 Neutral, 2 Somewhat tired, 4 Very tired, 0 No response, 1
Self-reported symptoms	Did you experience physical fatigue while using the AR glasses?	Not at all, 4 Minimal, 8 Neutral, 1 Somewhat tired, 1 Very tired, 0
Self-reported symptoms	Did you experience any headaches while using the AR glasses?	Not at all, 7 Minimal, 6 Neutral, 0 Somewhat, 1 Significantly, 0
Self-reported symptoms	Did you experience any dizziness while using the AR glasses?	Not at all, 7 Minimal, 6 Neutral, 1 Somewhat, 0 Significantly, 0
Self-reported symptoms	Did you experience any nausea while using the AR glasses?	Not at all, 7 Minimal, 6 Neutral, 0 Somewhat, 1 Significantly, 0
Adaptation and adoption	Would you recommend the use of AR glasses to other medical professionals?	Not at all, 1 Unlikely, 0 Neutral, 3 Somewhat, 7 Strongly, 3
Weight and fit	What did you think about the weight of the AR glasses?	Very light, 3 Slightly light, 1 Just right, 6 Slightly heavy, 3 Very heavy, 0 No response, 1
Visual usability	How was the visibility of patient information through the AR display?	Very hard to see, 0 Slightly hard to see, 8 Easy to see, 5 No response, 1
Visual usability	How was the visibility of the surrounding environment?	Very hard to see, 0 Slightly hard to see, 3 Easy to see, 10 No response, 1
Task interference	To what extent did you find the AR glasses interfered with clinical tasks?	Very obstructive, 0 Slightly obstructive, 3 Not obstructive, 10 No response, 1
Perceived responsiveness	How did you find the responsiveness of the facial recognition function?	Too slow, 0 Slightly slow, 2 Just right, 10 Slightly fast, 1 Too fast, 0 No response, 1

a AR: augmented reality.

### Perceived Clinical Utility and Future Adoption

For perceived assistance with clinical procedures, 5 of the 14 participants (36%) rated the device as “slightly helpful,” and 6 (43%) rated it as “helpful.” In total, 11 participants selected “slightly helpful” or “helpful” (79%; Wilson 95% CI 52%‐92%); the remaining 3 selected “neutral.” Similarly, for perceived support of clinical safety, 12 selected “slightly helpful” or “helpful” (86%; 95% CI 60%‐96%), with 3 (21%) selecting “slightly helpful” and 9 (64%) selecting “helpful”; 2 (14%; 95% CI 4%‐39%) selected “neutral.” Eleven of 14 participants expressed willingness to use the glasses in future practice (79%; 95% CI 52%‐92%), and 10 of 14 would recommend the prototype to other professionals (71%; 95% CI 45%‐88%).

### Wearability and Adaptation

of the 14 participants, 8 (57%; 95% CI 33%‐79%) reported that no or minimal time was needed to adapt to the glasses, 1 (7%; 95% CI 1%‐30%) selected “neutral,” 4 (29%; 95% CI 12%‐55%) selected “somewhat,” and 1 (7%; 95% CI 1%‐30%) selected “very much.” Among 13 respondents to the weight item, 3 (23%; 95% CI 8%‐50%) rated the glasses as very light, 1 (8%; 95% CI 1%‐33%) as slightly light, 6 (46%; 95% CI 23%‐71%) as just right, and 3 (23%; 95% CI 8%‐50%) as slightly heavy; 1 participant did not answer. In free-text comments, 4 of 5 participants (80%; 95% CI 38%‐96%) using prescription glasses mentioned difficulty wearing the prototype over their prescription glasses.

### Visual Usability, Task Interference, and Perceived Responsiveness

Patient information legibility was an important concern. Among 13 respondents, 8 (62%; 95% CI 36%‐82%) rated the displayed patient information as slightly hard to see, while 5 (38%; 95% CI 18%‐65%) rated it as easy to see; 1 participant did not answer. By contrast, 10 of 13 respondents (77%; 95% CI 50%‐92%) rated the surrounding environment as easy to see, and 3 (23%; 95% CI 8%‐50%) rated it as slightly hard to see. Ten of 13 (77%; 95% CI 50%‐92%) considered the device nonobstructive and 3 (23%; 95% CI 8%‐50%) as slightly obstructive. Perceived facial recognition responsiveness was rated as just right by 10 of 13 respondents (77%; 95% CI 50%‐92%), slightly slow by 2 (15%; 95% CI 4%‐42%), and slightly fast by 1 (8%; 95% CI 1%‐33%); 1 participant did not answer. These ratings do not constitute an objective latency or accuracy assessment.

### Self-Reported Symptoms

For eye fatigue, 5 of 13 respondents (38%; 95% CI 18%‐65%) selected “not at all,” 2 (15%; 95% CI 4%‐42%) selected “minimal,” 2 (15%; 95% CI 4%‐42%) selected “neutral,” and 4 (31%; 95% CI 13%‐58%) selected “somewhat tired”; none selected “very tired,” and 1 participant did not answer. Mild physical fatigue, headaches, and nausea were reported by a single participant. However, the reported symptoms were confined to the trial period and resolved spontaneously. No participant selected the highest-severity category for physical fatigue, headache, dizziness, or nausea.

## Discussion

### Principal Results

This study reported generally favorable reactions from doctors to the use of facial recognition–equipped AR glasses in a simulated clinical setting. Most participants considered the AR glasses to be useful both for performing medical procedures and in enhancing clinical safety.

Huang et al [6] reported favorable overall usability of an AR-AI assistant among 15 ophthalmic day-ward nurses, and Lin et al [7] evaluated an intelligent verification tool for ophthalmic surgical information among surgical nurses. To the best of our knowledge, this is the first study to evaluate the usability of AR glasses equipped with facial recognition functionality among physicians.

While no serious self-reported symptoms were observed in association with wearing the AR glasses, it is noteworthy that their use may induce mild symptoms, including eye fatigue, physical fatigue, headache, and nausea. Moreover, the AR glasses used in this study have room for technical improvement. Although the device used waveguide optics that enabled a more compact design compared to other AR display technologies [8] and achieved weight reduction through wired connection to a smartphone, 21% of participants rated it as “slightly heavy.” There is also strong anticipation of the development of wireless devices in the future. While this study only evaluated the facial recognition functionality of the AR glasses and limited the display to basic patient information, the device could potentially display a wider range of information, such as CT scans and real-time vital signs, during procedures. This could support doctors not only in outpatient care but also during surgery and other medical interventions. Further research is needed to evaluate the utility of AR glasses equipped with enhanced functions in clinical settings. Furthermore, AR glasses have potential applications for health care professionals beyond physicians. Our previous study [9] demonstrated that when scrub nurses used see-through head-mounted displays, it facilitated their understanding of surgical procedures and improved the efficiency of instrument handling. Additionally, since nurses and medical administrative staff are also involved in patient identification, implementing facial recognition technology could potentially benefit all medical staff. However, facial recognition in this setting should be regarded as an adjunct rather than a replacement for established patient identification procedures. Wristbands, barcodes or QR codes, and confirmation using multiple identifiers have the advantage of being embedded in established workflows. A near-eye system may offer hands-free access to contextual information and reduce the need to look away from the patient, but face matching can be affected by lighting, pose, occlusion, masks, image quality, database composition, demographic performance variation, and the selected decision threshold. A false match may also create automation bias. Therefore, practical clinical use requires a fail-safe system for unrecognized faces, as well as independent confirmation using standard identification methods.

### Limitations

This study was limited by a small sample size. Consequently, the 95% CIs for the survey responses are notably wide (eg, 52%‐92% for 11 of 14 participants), which underscores the substantial uncertainty in our point estimates. Although the prototype used a cloud-based facial recognition service, this study did not evaluate the data routing, retention, deletion, access control, or regulatory requirements necessary for clinical deployment. As this was a pilot usability study, precise device use duration, detailed facial data registration procedures, and database size were not recorded. The usability of the AR glasses was assessed subjectively via a questionnaire lacking objective indicators. Similarly, participants’ prior experience with AR technologies and familiarity with wearable devices were not recorded, which might have influenced individual adaptation times and usability ratings. Furthermore, the potential risk of cognitive overload associated with processing simultaneous real-world visual inputs and AR-displayed information was not evaluated in this study. Industry provision of the prototype and the participation of a company executive as an author may have introduced expectancy bias despite anonymous responses. Finally, no real patient clinical data were used; the displayed demographic and clinical information was fictitious. Because this study was conducted in a simulated setting rather than actual clinical practice, future research is needed to evaluate the system’s acceptability from the patients’ perspective.

### Conclusions

We found overall favorable responses from doctors regarding the use of AR glasses using waveguide optics with facial recognition functionality in a simulated clinical setting. This technology has the potential to assist medical professionals, providing a safeguard against patient misidentification.

## Supplementary material

10.2196/95664 Multimedia Appendix 1 Survey questionnaire on augmented reality glasses’ usability and potential adverse effects.

## Abbreviations

AR: augmented reality
