# Supplementary material for: Utility of Augmented Reality Glasses With Waveguide Optics and Facial Recognition Technology for Patient Verification in a Simulated Outpatient Setting: Physician Questionnaire Survey Study
Source: JMIR Hum Factors. 2026 Oct 2;13:e95664. doi: 10.2196/95664 (PMC13632967; doi:10.2196/95664)
Supplement: Supplementary file 1 [file humanfactors-v13-e95664-s001.docx]

| **Item** | **Questions** | **Ordered response options** |
| --- | --- | --- |
| 1 | Did the use of the AR glasses assist you in performing medical procedures? | Not at all; Slightly unhelpful; Neutral; Slightly helpful; Helpful |
| 2 | Did the use of the AR glasses help improve safety in clinical practice? | Not at all; Slightly unhelpful; Neutral; Slightly helpful; Helpful |
| 3 | Did it take time for you to get used to wearing the AR glasses? | Not at all; Minimal; Neutral; Somewhat; Very much |
| 4 | Do you wish to use AR glasses in future medical procedures? | Not at all; Unlikely; Neutral; Somewhat; Strongly |
| 5 | To what extent did you experience eye fatigue from using the AR glasses? | Not at all; Minimal; Neutral; Somewhat tired; Very tired |
| 6 | Did you experience physical fatigue while using the AR glasses? | Not at all; Minimal; Neutral; Somewhat tired; Very tired |
| 7 | Did you experience any headaches while using the AR glasses? | Not at all; Minimal; Neutral; Somewhat; Significantly |
| 8 | Did you experience any dizziness while using the AR glasses? | Not at all; Minimal; Neutral; Somewhat; Significantly |
| 9 | Did you experience any nausea while using the AR glasses? | Not at all; Minimal; Neutral; Somewhat; Significantly |
| 10 | Would you recommend the use of AR glasses to other medical professionals? | Not at all; Unlikely; Neutral; Somewhat; Strongly |
| 11 | What did you think about the weight of the AR glasses? | Very light; Slightly light; Just right; Slightly heavy; Very heavy |
| 12 | How was the visibility of patient information through the AR display? | Very hard to see; Slightly hard to see; Easy to see |
| 13 | How was the visibility of the surrounding environment? | Very hard to see; Slightly hard to see; Easy to see |
| 14 | To what extent did you find the AR glasses to interfere with clinical tasks? | Very obstructive; Slightly obstructive; Not obstructive |
| 15 | How did you find the responsiveness of the facial-recognition function? | Too slow; Slightly slow; Just right; Slightly fast; Too fast |

Please provide any additional comments regarding your experience with the AR glasses.
